# Association between arterial stiffness and autonomic dysfunction in participants underwent treadmill exercise testing: a cross-sectional analysis

**DOI:** 10.1038/s41598-024-53681-1

**Published:** 2024-02-13

**Authors:** Sungjoon Park, Hack-Lyoung Kim, Kyung-Taek Park, Hyun Sung Joh, Woo-Hyun Lim, Jae-Bin Seo, Sang-Hyun Kim, Myung-A Kim

**Affiliations:** 1 Division of Cardiology, Department of Internal Medicine, Boramae Medical Center, Seoul National University College of Medicine, 20 Boramae-ro 5-gil, Dongjak-gu, Seoul, 07061 Republic of Korea; 2 Department of Cardiology, Chung-Ang University Hospital, Seoul, Republic of Korea

**Keywords:** arterial stiffness, autonomic function, heart rate recovery, pulse wave velocity, treadmill exercise test, Cardiovascular biology, Atherosclerosis, Blood flow, Hypertension

## Abstract

Data on the impact of arterial stiffness on autonomic function are limited. We sought to investigate whether heart rate recovery (HRR), a predictor of autonomic function, is impaired in patients with increased arterial stiffness. A total of 475 participants (mean age 55.8 ± 11.1 years, 34.3% women) who underwent a treadmill exercise test (TET) for the evaluation of chest pain were retrospectively analyzed. All patients underwent brachial-ankle pulse wave velocity (baPWV) measurement on the same day. HRR was defined as the difference in heart rate from maximal exercise to 1 min of recovery. Participants with the lowest HRR tertile were older and had more cardiovascular risk factors than those with the highest HRR tertile. Simple correlation analysis showed that baPWV was negatively correlated with HRR (*r* =  − 0.327, *P* < 0.001). In multiple linear regression analysis, there was a significant association between baPWV and HRR, even after adjusting for potential confounders (*β* =  − 0.181, *P* < 0.001). In participants who underwent TET, baPWV was negatively correlated with HRR. The results of our study indicate a potential relationship between arterial stiffness and the autonomic nervous system.

## Introduction

Arterial stiffening is caused by the degradation of elastic fibers replaced by fibrosis, accumulation of lipids, migration of inflammatory cells, and proliferation of the smooth muscle cells of the arterial wall. This arterial remodeling process is accelerated by aging and other cardiovascular (CV) risk factors such as hypertension, diabetes, smoking, oxidative stress, and chronic inflammation^1,2^. It is important to understand the concept of arterial stiffness because it is associated with the occurrence of mortality and CV events regardless of existing traditional risk factors^3,4^. Consequently, information regarding arterial stiffness is primarily utilized in clinical settings to predict the subject’s CV prognosis. Among several methods of measuring arterial stiffness, the pulse wave velocity (PWV) is the most widely used due to its non-invasiveness, simplicity, and reproducibility^2^. Although carotid-femoral PWV (cfPWV) is considered the gold standard for arterial stiffness measurement among non-invasive methods^5^, the cfPWV measurement needs technical skill, and it may cause discomfort to the participants in finding carotid and femoral arteries^6^. On the other hand, brachial-ankle PWV (baPWV), developed later than cfPWV, is relatively simple to measure, takes less time, and has abundant clinical data^4,6,7^. Thus, baPWV may be more advantageous for risk stratification in mass screenings. Recently, baPWV has been widely used to evaluate CV risk among many participants, especially in Asian countries^8^.

Resting heart rate (HR), a critical cardiac output parameter, is an important clinical sign in evaluating the hemodynamic status^9^. The autonomic nervous system and catecholamines influence HR in response to dynamic exercise^10^. The increase in HR during exercise is mainly increased by vagal withdrawal at the beginning of the exercise. As HR reaches approximately 100 beats per minute or more, sympathetic activity further contributes to the rise. Following exercise termination, sympathetic nervous system activity rapidly decreases, and vagal tone is reactivated, leading to an exponential decrease in HR. Therefore, HR changes corresponding to different stages of exercise could serve as indicators of autonomic nervous system function^11,12^. Autonomic dysfunction is closely related to CVD, especially arrhythmia, so HR changes during exercise stress tests may be applied to predict CVD^13–15^. HR recovery (HRR) is defined as a difference between heart rate at peak exercise and within several minutes after resting during a treadmill exercise test (TET). HRR is known as a predictor of mortality independent from the treadmill exercise score^15,16^.

Although arterial stiffness and HRR are important indicators for predicting CVD, there are only a few studies on the correlation between these two indicators^17,18^. Knowing the relationship between these two variables is clinically significant because it can be applied to CV risk prediction or therapeutic development. Therefore, in this study, we aim to evaluate the association between baPWV and HRR in participants who underwent TET.

## Methods

### Study population

This single-center, retrospective study was conducted at the Boramae Medical Center in Seoul, Republic of Korea. The inclusion criteria for our study were adults aged 19 and older who presented with chest pain as their primary complaint during their initial visit to the center. These individuals underwent both TET and baPWV assessments on the same scheduled date, between January 2010 and November 2013. The measurement of baPWV was performed at the attending physician’s discretion as part of the CV evaluation. Participants with the following clinical conditions were excluded: (1) unstable vital signs, (2) left ventricular (LV) ejection fraction < 50%, (3) valvular regurgitation or stenosis of more than mild degree, (4) presence of regional wall motion abnormality of LV, (5) pericardial effusion, (6) uncontrolled arrhythmia, and (7) ankle-brachial index < 0.9 or > 1.4.

This study was performed in line with the principles of the Declaration of Helsinki. The Institutional Review Board (IRB) of Boramae Medical Center (Seoul, Korea) approved the study protocol and waived written informed consent due to the retrospective study design and the routine nature of the information collected (IRB number, 16-2013-167).

### Clinical data collection

Body mass index (BMI) was calculated as the ratio of the participants’ weight in kilograms divided by the height in meters squared (kg/m^2^). Using an oscillometric device, a trained nurse measured systolic BP (SBP) and diastolic BP (DBP). Hypertension was defined by a history of a hypertension diagnosis, SBP ≥ 140 mmHg, DBP ≥ 90 mmHg or current use of antihypertensive medications. Diabetes mellitus was defined by a history of the diagnosis of diabetes mellitus, fasting glucose level ≥ 126 mg/dL, glycated hemoglobin ≥ 6.5% or current use of antidiabetic medications. Dyslipidemia was defined as a history of dyslipidemia diagnosis, low-density lipoprotein cholesterol ≥ 160 mg/dL or current use of antidyslipidemic medications. Patients who smoked regularly within the past 12 months were considered current smokers. A previous history of coronary artery disease and stroke was defined based on documented clinical diagnosis by a cardiologist and neurologist, respectively. The diagnosis of coronary artery disease was restricted to myocardial infarction and coronary revascularization, including percutaneous coronary intervention and bypass surgery. Data on concomitant antihypertensive medications, including renin-angiotensin system blockers, calcium channel blockers, beta-blockers, diuretics, and statins, were also obtained. Laboratory tests were performed after an overnight fasting of 12 h. Serum levels of hemoglobin, glycated hemoglobin, glucose, creatinine, total cholesterol, low-density lipoprotein cholesterol, high-density lipoprotein, triglyceride, and high-sensitivity C-reactive protein were quantified. The estimated glomerular filtration rate (GFR) was calculated by the Modification of Diet in Renal Disease (MDRD) equation^19^. Transthoracic echocardiography was performed, and left ventricular ejection fraction was measured on the apical four- and two-chamber views using the biplane Simpson’s method.

### TET

A well-trained examiner conducted the TET using a commercially available device (GE healthcare CASE stress test system, Illinois, USA), following Bruce protocol^20^: the initial speed of the treadmill was 1.7 mile/h with a slope of 10°; the second speed was 2.5 mile/h with a slope of 12°; the third speed was 3.4 mile/h with slope 14°; and the fourth speed was 4.2 mile/hour with slope 16°^21^. Surface 12-lead electrocardiogram (ECG) was continuously monitored, and brachial BP was measured at rest, at the end of each stress stage, peak stress, and recovery stage. The speed accelerated every 3 min. All HR and BP were measured in a sitting position. Termination of the procedure was based on the American Heart Association criteria: (1) a drop in SBP of 10 mmHg or more from the baseline in the absence of other evidence of ischemia, (2) ST-segment elevation (> 1.0 mm) in leads without Q waves (other than V1 or aVR), (3) severe anginal pain at a level that the participant will wish to stop exercising or more serious, (4) central nervous system symptoms (e.g., ataxia, dizziness, or near syncope), (5) signs of poor perfusion (cyanosis or pallor), (6) sustained ventricular tachycardia, (7) technical difficulties monitoring the ECG or SBP, (8) participants’ request to stop^22^. Exercise capacity is the assessment of exercise performance quantified by metabolic equivalent tasks (METs). Exercise duration is defined as the total exercise time expressed as a second (s). ST segment change and positive final diagnosis are determined by the magnitude of exercise-induced ST displacement. ST segment change is specified as a horizontal or down-sloping ST deviation greater than 0.5 or 1.0 mm at 60 ms after the J point compared to the P-Q junction. Based on Duke score, which is an Arabic number composed of chest pain and ST depression, it is classified as high, intermediate, and low risk. These values correspond to low-risk (with a score of ≥  + 5), moderate-risk (with scores ranging from − 10 to + 4), and high-risk (with a score of ≤  − 11). After exercise termination, participants rested in a sitting position, and HR and BP were measured at 1 min and 2 min after the exercise. HRR was defined as the difference between the maximum HR during exercise and the HR 1 min after the test (HRR1). Although the difference between the maximum HR during exercise and the HR 2 min post-test (HRR2) displayed a correlation strength with baPWV comparable to HRR1 in univariable analysis (Supplementary Fig. S1), HRR1 exhibited a stronger correlation with baPWV in multivariable analysis (Supplementary Tables S1, S2). Based on the prior studies that identify BP response post-exercise as a crucial marker of autonomic function^23,24^, we evaluated the changes in SBP and DBP at 2 and 3 min after the TET termination, comparing them to the peak BP levels observed during the TET.

### baPWV measurement

The baPWV measurements were performed on the same day as TET. The volume-plethysmographic apparatus (VP-2000; Colin Co., Ltd., Komaki, Japan) was used to measure baPWV. Caffeine, alcohol consumption, and cigarette smoking were prohibited at least 12 h before the measurement. Patients were examined after resting in a quiet room in the supine position for a minimum of 5 min. Electrocardiographic electrodes were placed on both wrists, phonocardiographic electrodes were placed on the edge of the sternum to detect heart sounds, and pneumatic cuffs were wrapped around the upper arms and ankles. PWV value was calculated as a distance divided by transit time with the distance between measurement points estimated based on participant height^25^. Transit time was derived from the start point of the brachial pulse wave to the start of the ankle pulse wave. The average value of left and right baPWV measurements was used for the study. A single well-trained staff member conducted the baPWV measurements. In our laboratory, the coefficient of variance for inter-observer reliability of baPWV was 5.1% in our laboratory^26^.

### Statistical analysis

Continuous variables are presented as the mean ± standard deviation, while categorical variables are expressed as percentages. Participants were categorized into three groups based on HRR tertiles. The clinical characteristics of these groups were compared using analysis of variance for continuous variables and the Chi-square test for categorical variables. To explore univariable associations between two continuous variables, Pearson’s bivariate correlation analysis was employed. Additionally, the study used multivariable linear regression analysis to investigate the independent association between baPWV and HRR. This analysis included all variables that showed statistical significance in the univariable analyses: sex, BMI, smoking status, high-density lipoprotein cholesterol, exercise capacity, SBP change at 3 min after exercise and resting and peak exercise HR. These potential confounders were controlled in the multivariable model using the forward selection method to ensure the robustness of the findings. To assess multicollinearity among explanatory variables, the variance inflation factor (VIF) was introduced. Multiple binary logistic regression analyses were conducted to evaluate whether a higher baPWV (≥ 1400 cm/s), indicative of abnormal arterial stiffening^8^ could predict lower HRR (< 26 beats/min, the median value). The same confounders were controlled for as those used in the multiple linear regression analysis. In the multivariable analyses, we evaluated model fit by comparing the changes in Akaike information criterion (AIC) and Bayesian information criterion (BIC) values that occurred upon adding baPWV as an independent variable. To visually represent and illustrate linear correlations between baPWV and HRR, scatter plots were utilized. Statistical significance was determined at a *P* value of less than 0.05. All statistical analyses were conducted using the SPSS 25.0 software (IBM Corp., Armonk, NY, USA).

## Results

**Figure 1:**
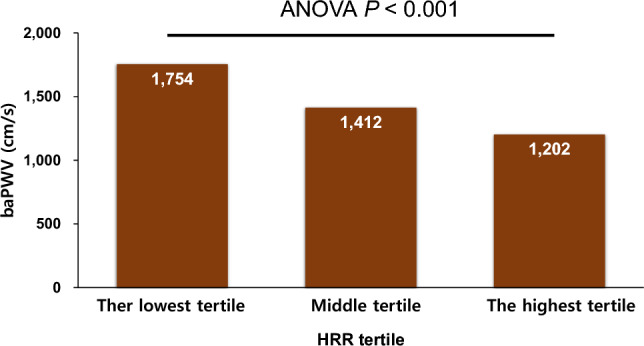
baPWV values according to the HRR tertile. *baPWV* brachial-ankle pulse wave velocity, *HRR* heart rate recovery, *ANOVA* analysis of variance.

**Figure 2:**
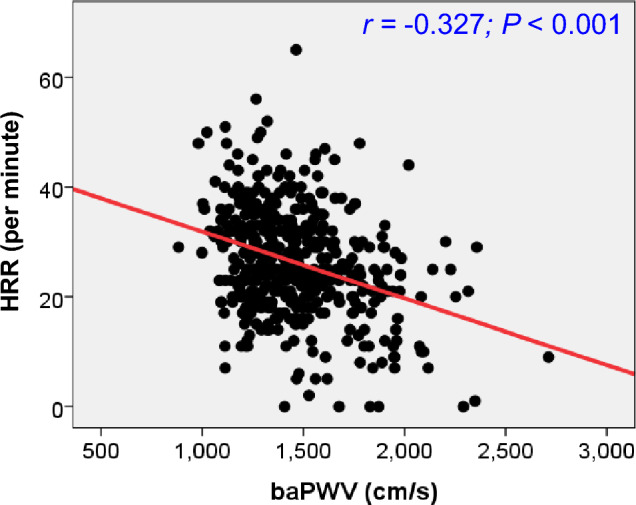
Linear correlation between baPWV and HRR. *baPWV* brachial-ankle pulse wave velocity, *HRR* heart rate recovery.

A total of 475 participants were analyzed. Two hundred and fifty-seven (54.1%) individuals completed the TET by achieving the target heart rate. The most common reason for early termination of TET was ST changes in 104 (21.9%) cases, followed by chest pain in 48 (10.1%) cases. The clinical characteristics of the study participants according to HRR tertile are presented in Table 1. The mean age was 55.8 ± 11.1 years, and 34.3% were female. Lower HRR was associated with more advanced age and a lower proportion of females compared to a higher HRR. Compared to participants with higher HRR, participants with lower HRR exhibited more CV risk factors, including a greater BMI, a higher BP, and the higher prevalence of hypertension, diabetes mellitus, cigarette smoking, and a previous history of coronary artery disease. CV medications, such as calcium channel blockers, beta-blockers, diuretics, and statins, were more frequently prescribed in participants with lower HRR than those with higher HRR. In laboratory findings, the blood levels of glucose, glycated hemoglobin, and creatinine were higher, and the blood levels of high-density lipoprotein cholesterol were lower in participants with lower HRR than those with higher HRR. Figure 1 demonstrates baPWV values according to HRR tertiles. baPWV gradually increased from the lowest to the highest tertile of HRR (baPWV: the lowest 1754 ± 207 cm/s, middle 1412 ± 61 cm/s, the highest 1202 ± 78 cm/s; *P* < 0.001). Table 2 shows TET results according to the HRR tertiles. Lower HRR was associated with decreased exercise capacity, duration, SBP at three minutes after exercise, and peak exercise HR. In Pearson’s correlation analysis, baPWV negatively correlated with HRR (*r* =  − 0.327, *P* < 0.001). Figure 2 shows the linear relationship between baPWV and HRR. Table 3 shows the results of multiple linear regression analysis of factors associated with HRR. There was a significant association between baPWV and HRR even after adjusting for potential confounders (*β* =  − 0.181, *P* < 0.001). Multiple binary logistic regression model showed a similar finding that a higher baPWV ≥ 1400 cm/s was independently associated with a lower HRR (< 26 beats/min, the median value) (odds ratio, 1.92; 95% confidence interval 1.19–3.11; *P* = 0.007) (Table 4). The inclusion of baPWV in the multivariable analyses resulted in a reduction of both AIC and BIC values in both multiple linear regression (AIC, 2492 to 2480; BIC, 2531 to 2522) and logistic regression analyses (AIC, 452 to 447; BIC, 487 to 486). This indicates that models incorporating baPWV demonstrate a better fit. Female sex, BMI, current smoker, SBP change at 3 min after exercise, resting HR, and peak exercise HR were other significant risk factors associated with HRR in both multivariable models (*P* < 0.05 for each).

**Table 1:** Clinical characteristics of study participants according to HRR tertile.

Characteristic	HRR tertile			*P*
	The lowest (0–22 beats/min) (n = 161)	Middle (23–30 beats/min) (n = 156)	The highest (31–65 beats/min) (n = 158)	
Age (years)	58.5 ± 11.1	56.1 ± 10.7	52.8 ± 10.7	< 0.001
Female sex	40 (24.8)	56 (35.8)	67 (42.4)	0.004
Body mass index (kg/m^2^)	25.2 ± 3.3	24.8 ± 3.4	24.2 ± 2.8	0.020
Systolic BP (mmHg)	129.5 ± 14.7	126.2 ± 15.2	119.9 ± 15.5	< 0.001
Diastolic BP (mmHg)	78.7 ± 9.0	76.2 ± 9.8	73.4 ± 10.6	< 0.001
Cardiovascular risk factors				
Hypertension	90 (55.9)	70 (44.8)	57 (36.0)	0.002
Diabetes mellitus	38 (23.6)	17 (10.9)	17 (10.7)	0.001
Dyslipidemia	61 (37.8)	56 (35.8)	32 (20.2)	0.001
Current smoker	43 (26.7)	29 (18.5)	21 (13.2)	0.010
Coronary artery disease	34 (21.1)	31 (19.8)	10 (6.3)	< 0.001
Stroke	3 (1.9)	1 (0.6)	0	0.180
Cardiovascular medications				
RAS inhibitors	63 (39.1)	48 (30.1)	33 (20.9)	0.002
Calcium channel blockers	70 (43.5)	47 (29.7)	23 (14.6)	< 0.001
Beta-blockers	51 (31.6)	46 (29.4)	16 (10.1)	< 0.001
Diuretics	18 (11.1)	15 (9.6)	3 (1.9)	0.004
Statin	88 (54.7)	77 (49.4)	47 (29.7)	< 0.001
Laboratory findings				
Hemoglobin (g/dL)	14.4 ± 2.1	14.2 ± 1.6	14.2 ± 1.4	0.717
Glucose (mg/dL)	115.6 ± 44.2	107.6 ± 27.7	99.5 ± 18.4	< 0.001
Glycated hemoglobin (%)	6.2 ± 1.2	5.9 ± 0.9	5.7 ± 0.7	0.001
Creatinine (mg/dL)	0.96 ± 0.25	0.87 ± 0.18	0.85 ± 0.19	< 0.001
GFR (mL/min/1.73 m^2^)	81.2 ± 18.6	86.9 ± 17.0	90.4 ± 21.8	< 0.001
Total cholesterol (mg/dL)	183.7 ± 40.0	187.0 ± 47.2	189.8 ± 35.6	0.426
LDL cholesterol (mg/dL)	113.9 ± 38.4	116.3 ± 38.4	118.4 ± 31.7	0.587
HDL cholesterol (mg/dL)	47.6 ± 11.3	48.5 ± 11.0	51.0 ± 11.5	0.031
Triglyceride (mg/dL)	130.4 ± 73.7	127.9 ± 79.9	113.8 ± 65.9	0.117
C-reactive protein (mg/dL)	0.26 ± 0.72	0.21 ± 0.58	0.15 ± 0.39	0.395
LV ejection fraction (%)	66.9 ± 5.9	67.4 ± 5.7	66.1 ± 7.4	0.301

Numbers are expressed as mean ± standard deviation or n (%).

*HRR* heart rate recovery, *BP* blood pressure, *RAS* renin-angiotensin system, *GFR* glomerular filtration rate, *LDL* low-density lipoprotein, *HDL* high-density lipoprotein, *LV* left ventricular.

**Table 2:** The results of treadmill exercise test according to HRR tertile.

Result variable	HRR tertile			*P*
	The lowest (0–22 beats/min) (n = 161)	Middle (23–30 beats/min) (n = 156)	The highest (31–65 beats/min) (n = 158)	
Exercise capacity, METs	10.4 ± 2.6	10.9 ± 2.6	11.4 ± 1.8	0.010
Exercise duration (s)	526 ± 148	551 ± 113	586 ± 97	< 0.001
Chest pain, yes	22 (13.7)	17 (10.9)	9 (5.7)	0.057
ST segment change, yes	38 (23.6)	36 (23.1)	30 (19.0)	0.553
Positive final diagnosis, yes	40 (24.8)	32 (20.5)	23 (14.6)	0.097
Resting SBP (mmHg)	129 ± 17	133 ± 18	124 ± 17	< 0.001
Peak exercise SBP (mmHg)	171 ± 26	176 ± 26	171 ± 25	0.157
SBP change at 2 min after exercise (mmHg)	12.1 ± 14.0	9.4 ± 13.9	7.7 ± 13.9	0.020
SBP change at 3 min after exercise (mmHg)	16.0 ± 16.4	19.7 ± 16.3	23.6 ± 17.0	< 0.001
Resting DBP (mmHg)	83.5 ± 12.0	85.1 ± 10.3	83.4 ± 12	0.416
Peak exercise DBP (mmHg)	70.0 ± 43.6	66.5 ± 48.2	63.2 ± 47.3	0.420
DBP change at 2 min after exercise (mmHg)	11.6 ± 11.7	12.2 ± 14.1	11.1 ± 15.5	0.804
DBP at change 3 min after exercise (mmHg)	12.0 ± 15.4	14.2 ± 18.0	13.8 ± 17.9	0.514
Resting HR (beats/min)	80.2 ± 13.4	77.4 ± 13.8	73.9 ± 12	0.001
Peak exercise HR (beats/min)	139.7 ± 21.8	147.3 ± 20.1	153.2 ± 16.0	< 0.001
HRR (beats/min)	16.0 ± 5.5	26.2 ± 2.4	36.9 ± 5.6	< 0.001

Numbers are expressed as mean ± standard deviation or n (%).

*HRR* heart rate recovery, *METs* metabolic equivalent tasks, *SBP* systolic blood pressure, *DBP* diastolic blood pressure, *HR* heart rate.

**Table 3:** Multivariable linear regression analysis showing independent predictors for HRR.

Variable	*β*	*P*	VIF
Female sex, yes	0.153	0.001	1.244
Body mass index (kg/m^2^)	− 0.166	< 0.001	1.087
Current smoker, yes	− 0.160	< 0.001	1.125
High-density lipoprotein cholesterol (mg/dL)	0.083	0.060	1.137
Exercise capacity, METs	− 0.145	0.006	1.599
SBP change at 3 min after exercise (mmHg)	0.181	< 0.001	1.026
Resting HR (beats/min)	− 0.447	< 0.001	1.590
Peak exercise HR (beats/min)	0.512	< 0.001	1.842
baPWV (cm/s)	− 0.181	< 0.001	1.174

*HRR* heart rate recovery, *VIF* variance inflation factor, *METs* metabolic equivalent tasks, *SBP* systolic blood pressure, *HR* heart rate, *baPWV* brachial-ankle pulse wave velocity.

**Table 4:** Multiple binary logistic regression analysis showing independent predictors for lower HRR (< 26 beats/min, the median value).

Variable	OR (95% CI)	*P*
Female sex, yes	0.63 (0.37–1.05)	0.080
Body mass index ≥ 25 kg/m^2^	2.01 (1.25–3.22)	0.004
Current smoker, yes	3.31 (1.75–6.23)	< 0.001
HDL cholesterol < 40 mg/dL	1.99 (1.05–3.75)	0.033
Exercise capacity (METs), < median	0.89 (0.54–1.47)	0.663
SBP change at 3 min after exercise (mmHg), < median	0.50 (0.31–0.80)	0.004
Resting HR (beats/min), < median	2.65 (1.55–4.53)	< 0.001
Peak exercise HR (beats/min), < median	0.49 (0.29–0.83)	0.009
baPWV ≥ 1400 cm/s	1.92 (1.19–3.11)	0.007

*HRR* heart rate recovery, *OR* odds ratio, *CI* confidence interval, *HDL* high-density lipoprotein, *METs* metabolic equivalent tasks, *SBP* systolic blood pressure, *HR* heart rate, *baPWV* brachial-ankle pulse wave velocity.

## Discussion

This study demonstrated that increased baPWV was independently associated with attenuated HRR even after adjusting potential confounders in patients undergoing TET. In addition, other factors such as sex, obesity, smoking, recovery of SBP, resting HR, and peak exercise HR were related to attenuated HRR in multivariable analyses.

The regulation of the autonomic nerve system is crucial for maintaining the homeostasis of the CV system, particularly in controlling HR. During the intensive exercise, the parasympathetic nerve is withdrawn, and the sympathetic nerve is activated, leading to increased HR. After termination of exercise, HR exponentially decreases, primarily due to the predominance of the parasympathetic nerve over sympathetic withdrawal^11,12^. HRR is a reliable and reproducible parameter that reflects immediate alterations in autonomic nerve function after the termination of exercise^27^. Of note, it has been shown that slow HRR is associated with the severity of coronary artery disease in participants without traditional CV risk factors^28,29^. Furthermore, it predicts future CV morbidity and mortality^14,15^. Therefore, identifying clinical factors associated with HRR is meaningful for understanding the underlying pathophysiology and developing preventive or therapeutic strategies, especially among individuals at high coronary risk.

It has been suggested that HRR is associated with various CV risk factors, such as older age, smoking, chronic kidney disease, and metabolic syndrome^30,31^. Several studies have reported a correlation between arterial stiffness and HRR^17,18^. In a study of 209 healthy adults in the United States, aortic pulse wave velocity measured by magnetic resonance imaging was found to be significantly correlated with HRR^18^. Another Korean study of 154 normotensive patients without overt atherosclerosis demonstrated a significant association between baPWV and HRR^17^. In both studies, similar to ours, HRR was measured at 1 min after exercise. While these results were obtained from small studies, these findings are also in line with our findings, showing the association between arterial stiffness and HRR. Our data furnishes additional evidence regarding the pivotal role of arterial stiffness in the context of cardiac autonomic dysfunction.

Although the precise underlying pathophysiology of the association between arterial stiffness and HRR has yet to be elucidated, several hypotheses can be proposed. For instance, arterial stiffness might induce autonomic dysfunction through blunted baroreflex sensitivity^32–34^. The baroreceptor is located in the carotid sinus and detects stretching of the aortic arch as BP increases. Arterial stiffening deforms the vascular wall components such as elastin, collagen, and vascular smooth muscle cells in baroreceptors of the aortic arch and carotid sinus. Arterial stiffening also causes carotid sinus hypertrophy and subsequently attenuates baroreceptor sensitivity, which detects fluctuation of intravascular pressure^35^.

### Clinical implications

For the proper clinical diagnosis and treatment of particular diseases, understanding the underlying pathophysiology is essential. While the function of the autonomic nervous system is crucial in the CV system, but the regulation of its function remains incompletely understood. Our study showed that HRR was associated with baPWV in participants who underwent TET, providing additional evidence on the possible role of arterial stiffness in regulating CV autonomic function. In addition, CV autonomic dysfunction may be suggested as another possible mechanism for the prognostic value of arterial stiffness in increasing the risk of CV events. Moreover, since baPWV is a noninvasive and simple measure, it can be used as a screening or monitoring tool for CV autonomic abnormality in high-risk individuals. Further studies are needed to determine whether methods that improve arterial stiffness can also improve HRR.

### Study limitations

In addition to the retrospective design, there are several limitations of this study. First, our study did not confirm the causal relationship between baPWV and HRR, because of cross-sectional analysis. Moreover, demonstrating the association between baPWV and HRR does not necessarily imply a direct correlation between the results of baPWV and cardiac autonomic function. Second, we enrolled patients undergoing TET to evaluate chest discomfort; thus, we should be cautious when applying our results to other participant groups. Finally, while cfPWV is considered a golden standard non-invasive method for measuring arterial stiffness^5^, we conducted only baPWV measurements. However, it has been reported that baPWV correlated well with cfPWV^36^ and invasive data^24^. Additionally, the clinical values of baPWV have been shown in many clinical studies^4,6,8^. Moreover, baPWV is more suitable as a screening tool because it is simpler and more convenient to measure than cfPWV^6^.

## Conclusions

baPWV was negatively correlated with HRR in subjects who underwent TET. This result suggests a possible role of arterial stiffness in impaired cardiac autonomic response during exercise.

### Supplementary Information

Supplementary Information.

## Data Availability

All data generated or analyzed during this study are included in this published article.
