# Supplementary material for: Association between arterial stiffness and autonomic dysfunction in participants underwent treadmill exercise testing: a cross-sectional analysis
Source: Sci Rep. 2024 Feb 13;14:3588. doi: 10.1038/s41598-024-53681-1 (PMC10864279; doi:10.1038/s41598-024-53681-1)
Supplement: Supplementary file 1 — Supplementary Information. [file 41598_2024_53681_MOESM1_ESM.docx]

**Supplementary data**

**Supplementary Figure S1. Associations of baPWV with various autonomic function parameters of TET**


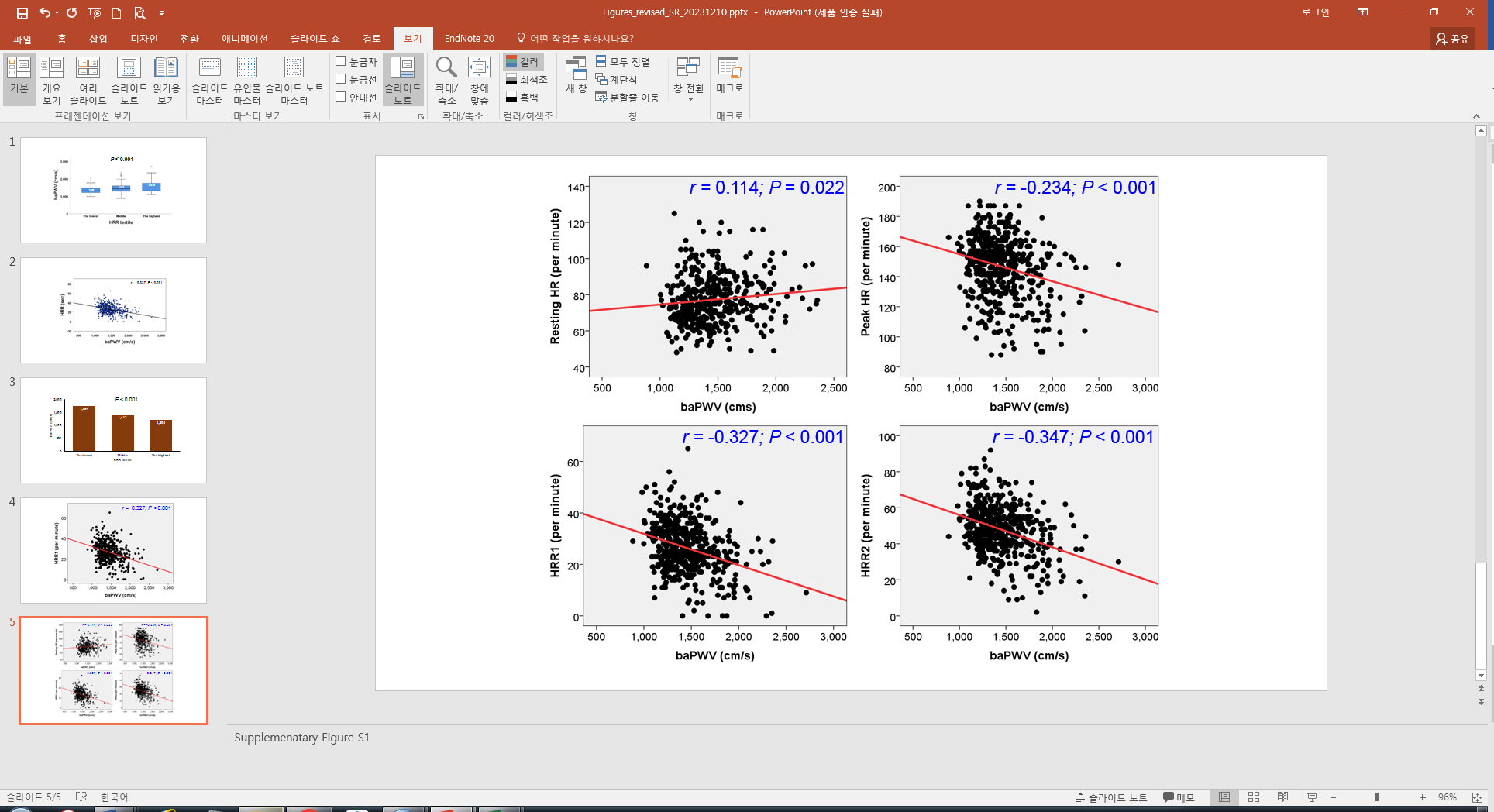


baPWV, brachial-ankle pulse wave velocity; TET, treadmill exercise test; HR, heart rate; HRR1, heart rate recovery at 1 minute; HRR2, heart rate recovery at 2 minute.

**Supplementary Table S1. Factors associated with HRR1 in linear regression analyses**

| **Variables** | **B** | **SE** | ***β*** | **t** | ***P*** | **tolerance** | **VIF** |
| --- | --- | --- | --- | --- | --- | --- | --- |
| intercept | 43.6600 | 2.3355 |  | 18.694 | 0.000 |  |  |
| baPWV | -0.0116 | 0.0015 | -0.327 | -7.549 | 0.000 | 1.000 | 1.000 |
| intercept | 54.5407 | 4.1684 |  | 13.084 | 0.000 |  |  |
| baPWV | -0.0093 | 0.0018 | -0.263 | -5.233 | 0.000 | 0.705 | 1.418 |
| Age | -0.1110 | 0.0450 | -0.125 | -2.465 | 0.014 | 0.689 | 1.452 |
| Female | 3.8703 | 0.8817 | 0.188 | 4.390 | 0.000 | 0.976 | 1.025 |
| BMI | -0.3815 | 0.1299 | -0.125 | -2.937 | 0.003 | 0.988 | 1.012 |
| intercept | 51.9348 | 4.4038 |  | 11.793 | 0.000 |  |  |
| baPWV | -0.0074 | 0.0018 | -0.210 | -4.076 | 0.000 | 0.638 | 1.568 |
| Age | -0.0737 | 0.0461 | -0.083 | -1.596 | 0.111 | 0.621 | 1.612 |
| Female | 2.4136 | 0.9256 | 0.117 | 2.608 | 0.009 | 0.838 | 1.194 |
| BMI | -0.3748 | 0.1313 | -0.123 | -2.854 | 0.005 | 0.914 | 1.094 |
| HTN | 0.7636 | 1.0151 | 0.039 | 0.752 | 0.452 | 0.631 | 1.584 |
| DM | -2.5272 | 1.2095 | -0.093 | -2.089 | 0.037 | 0.857 | 1.168 |
| Dyslipidemia | -0.2542 | 1.0451 | -0.012 | -0.243 | 0.808 | 0.686 | 1.458 |
| Stroke | -3.1052 | 4.6447 | -0.029 | -0.669 | 0.504 | 0.894 | 1.118 |
| CAD | -0.8480 | 1.3815 | -0.032 | -0.614 | 0.540 | 0.635 | 1.575 |
| CKD | -3.0308 | 2.5016 | -0.054 | -1.212 | 0.226 | 0.842 | 1.188 |
| Current smoker | -3.4713 | 1.0859 | -0.141 | -3.197 | 0.001 | 0.868 | 1.152 |
| RAS blockers | 1.9555 | 1.2498 | 0.092 | 1.565 | 0.118 | 0.489 | 2.047 |
| CCB | -3.8190 | 1.1639 | -0.178 | -3.281 | 0.001 | 0.573 | 1.746 |
| βB | -1.7828 | 1.1926 | -0.078 | -1.495 | 0.136 | 0.625 | 1.600 |
| Diuretics | -2.0337 | 1.7213 | -0.055 | -1.181 | 0.238 | 0.776 | 1.288 |
| Statins | 0.4387 | 1.1670 | 0.022 | 0.376 | 0.707 | 0.479 | 2.086 |
| intercept | 50.6459 | 4.3040 |  | 11.767 | 0.000 |  |  |
| baPWV | -0.0076 | 0.0018 | -0.213 | -4.211 | 0.000 | 0.660 | 1.515 |
| Age | -0.0699 | 0.0450 | -0.079 | -1.553 | 0.121 | 0.652 | 1.533 |
| Female | 2.6396 | 0.9033 | 0.128 | 2.922 | 0.004 | 0.879 | 1.137 |
| BMI | -0.3127 | 0.1282 | -0.102 | -2.439 | 0.015 | 0.959 | 1.043 |
| DM | -2.5163 | 1.1765 | -0.092 | -2.139 | 0.033 | 0.905 | 1.105 |
| Current smoker | -3.1841 | 1.0693 | -0.129 | -2.978 | 0.003 | 0.895 | 1.117 |
| CCB | -3.1079 | 0.9688 | -0.145 | -3.208 | 0.001 | 0.826 | 1.210 |
| βB | -1.7161 | 1.0262 | -0.075 | -1.672 | 0.095 | 0.844 | 1.185 |
| intercept | 33.5160 | 12.2327 |  | 2.740 | 0.007 |  |  |
| baPWV | -0.0113 | 0.0025 | -0.310 | -4.449 | 0.000 | 0.603 | 1.659 |
| Age | -0.1003 | 0.0747 | -0.098 | -1.344 | 0.180 | 0.554 | 1.804 |
| Female | 4.5475 | 1.5126 | 0.219 | 3.006 | 0.003 | 0.552 | 1.812 |
| BMI | -0.1848 | 0.1772 | -0.063 | -1.043 | 0.298 | 0.794 | 1.260 |
| DM | -0.8206 | 2.0783 | -0.029 | -0.395 | 0.693 | 0.527 | 1.897 |
| Current smoker | -3.7571 | 1.5349 | -0.152 | -2.448 | 0.015 | 0.762 | 1.312 |
| CCB | -3.5509 | 1.4298 | -0.155 | -2.483 | 0.014 | 0.754 | 1.326 |
| βB | 0.1939 | 1.6102 | 0.008 | 0.120 | 0.904 | 0.714 | 1.400 |
| Hb | 0.9069 | 0.4914 | 0.140 | 1.846 | 0.066 | 0.508 | 1.970 |
| HbA1c | 0.2127 | 0.8498 | 0.020 | 0.250 | 0.803 | 0.479 | 2.089 |
| GFR | -0.0109 | 0.0336 | -0.020 | -0.324 | 0.746 | 0.742 | 1.348 |
| LDL-C | -0.0003 | 0.0159 | -0.001 | -0.022 | 0.983 | 0.887 | 1.127 |
| HDL-C | 0.1175 | 0.0529 | 0.138 | 2.219 | 0.027 | 0.756 | 1.322 |
| TG | 0.0025 | 0.0087 | 0.018 | 0.284 | 0.777 | 0.762 | 1.312 |
| CRP | 0.5191 | 1.8400 | 0.016 | 0.282 | 0.778 | 0.873 | 1.145 |
| intercept | 50.0118 | 5.5262 |  | 9.050 | 0.000 |  |  |
| baPWV | -0.0087 | 0.0019 | -0.241 | -4.624 | 0.000 | 0.658 | 1.521 |
| Age | -0.0670 | 0.0502 | -0.071 | -1.336 | 0.182 | 0.637 | 1.569 |
| Female | 2.2905 | 0.9504 | 0.110 | 2.410 | 0.016 | 0.858 | 1.166 |
| BMI | -0.3139 | 0.1380 | -0.102 | -2.275 | 0.023 | 0.894 | 1.118 |
| DM | -2.4539 | 1.2152 | -0.090 | -2.019 | 0.044 | 0.892 | 1.121 |
| Current smoker | -3.8457 | 1.1168 | -0.155 | -3.444 | 0.001 | 0.881 | 1.135 |
| CCB | -2.7968 | 0.9911 | -0.131 | -2.822 | 0.005 | 0.836 | 1.197 |
| βB | -1.8683 | 1.0588 | -0.081 | -1.765 | 0.078 | 0.840 | 1.190 |
| HDL | 0.0465 | 0.0395 | 0.053 | 1.177 | 0.240 | 0.870 | 1.149 |
| intercept | 53.3988 | 8.5329 |  | 6.258 | 0.000 |  |  |
| baPWV | -0.0075 | 0.0020 | -0.216 | -3.707 | 0.000 | 0.643 | 1.554 |
| Age | -0.0874 | 0.0562 | -0.102 | -1.555 | 0.121 | 0.514 | 1.944 |
| Female | 3.0005 | 1.0961 | 0.145 | 2.737 | 0.007 | 0.785 | 1.273 |
| BMI | -0.3439 | 0.1523 | -0.117 | -2.258 | 0.025 | 0.818 | 1.223 |
| DM | -1.5564 | 1.4649 | -0.053 | -1.062 | 0.289 | 0.873 | 1.145 |
| Current smoker | -3.2575 | 1.2130 | -0.136 | -2.686 | 0.008 | 0.852 | 1.174 |
| CCB | -3.2564 | 1.1431 | -0.146 | -2.849 | 0.005 | 0.840 | 1.191 |
| βB | -1.3289 | 1.2037 | -0.056 | -1.104 | 0.270 | 0.857 | 1.167 |
| LVEF | -0.1455 | 0.0756 | -0.093 | -1.924 | 0.055 | 0.945 | 1.058 |
| LVMI | 0.0516 | 0.0245 | 0.109 | 2.109 | 0.036 | 0.816 | 1.225 |
| septal e' | 38.3041 | 27.8463 | 0.096 | 1.376 | 0.170 | 0.451 | 2.219 |
| e/e' | 0.0910 | 0.2446 | 0.025 | 0.372 | 0.710 | 0.481 | 2.079 |
| intercept | 51.7692 | 4.5793 |  | 11.305 | 0.000 |  |  |
| baPWV | -0.0083 | 0.0019 | -0.239 | -4.469 | 0.000 | 0.701 | 1.426 |
| Age | -0.1161 | 0.0473 | -0.134 | -2.453 | 0.015 | 0.671 | 1.491 |
| Female | 3.1712 | 0.9739 | 0.156 | 3.256 | 0.001 | 0.876 | 1.142 |
| BMI | -0.4058 | 0.1345 | -0.139 | -3.016 | 0.003 | 0.945 | 1.059 |
| Current smoker | -2.9789 | 1.1510 | -0.124 | -2.588 | 0.010 | 0.873 | 1.145 |
| CCB | -3.5692 | 1.0529 | -0.162 | -3.390 | 0.001 | 0.879 | 1.138 |
| LVMI | 0.0447 | 0.0225 | 0.095 | 1.987 | 0.048 | 0.873 | 1.145 |

HRR1, heart rate recovery at 1 minute; baPWV, brachial-ankle pulse wave velocity; SE, standard error; VIF, variance inflation factor; BMI, body mass index; DM, diabetes mellitus; CCB, calcium channel blocker; βB, β-blocker; Hb, hemoglobin; GFR, glomerular filtration rate; LDL-C, low-density lipoprotein cholesterol; HDL-C, high-density lipoprotein cholesterol; TG, triglyceride; CRP, C-reactive protein; LVMI, left ventricular mass index.

**Supplementary Table S2. Factors associated with HRR2 in linear regression analyses**

| **Variables** | **B** | **SE** | **β** | **t** | ***P*** | **tolerance** | **VIF** |
| --- | --- | --- | --- | --- | --- | --- | --- |
| intercept | 73.5135 | 3.3785 |  | 21.759 | 0.000 |  |  |
| baPWV | -0.0171 | 0.0022 | -0.347 | -7.680 | 0.000 | 1.000 | 1.000 |
| intercept | 86.1175 | 5.9571 |  | 14.456 | 0.000 |  |  |
| baPWV | -0.0103 | 0.0026 | -0.200 | -4.025 | 0.000 | 0.705 | 1.418 |
| Age | -0.3246 | 0.0644 | -0.253 | -5.043 | 0.000 | 0.689 | 1.452 |
| Female | 6.0265 | 1.2600 | 0.202 | 4.783 | 0.000 | 0.976 | 1.025 |
| BMI | -0.2739 | 0.1856 | -0.062 | -1.476 | 0.141 | 0.988 | 1.012 |
| intercept | 77.4612 | 6.2510 |  | 12.392 | 0.000 |  |  |
| baPWV | -0.0066 | 0.0026 | -0.128 | -2.547 | 0.011 | 0.638 | 1.568 |
| Age | -0.2339 | 0.0655 | -0.182 | -3.571 | 0.000 | 0.621 | 1.612 |
| Female | 4.2574 | 1.3138 | 0.142 | 3.240 | 0.001 | 0.838 | 1.194 |
| BMI | -0.1833 | 0.1864 | -0.041 | -0.983 | 0.326 | 0.914 | 1.094 |
| HTN | 0.6722 | 1.4409 | 0.024 | 0.467 | 0.641 | 0.631 | 1.584 |
| DM | -2.5974 | 1.7169 | -0.066 | -1.513 | 0.131 | 0.857 | 1.168 |
| Dyslipidemia | -0.8826 | 1.4835 | -0.029 | -0.595 | 0.552 | 0.686 | 1.458 |
| Stroke | -1.3991 | 6.5930 | -0.009 | -0.212 | 0.832 | 0.894 | 1.118 |
| CAD | -1.6411 | 1.9609 | -0.042 | -0.837 | 0.403 | 0.635 | 1.575 |
| CKD | -6.2725 | 3.5509 | -0.077 | -1.766 | 0.078 | 0.842 | 1.188 |
| Current smoker | -2.5040 | 1.5414 | -0.070 | -1.625 | 0.105 | 0.868 | 1.152 |
| RAS blockers | 1.6806 | 1.7740 | 0.055 | 0.947 | 0.344 | 0.489 | 2.047 |
| CCB | -5.7329 | 1.6521 | -0.184 | -3.470 | 0.001 | 0.573 | 1.746 |
| βB | -3.1032 | 1.6929 | -0.093 | -1.833 | 0.067 | 0.625 | 1.600 |
| Diuretics | -2.4234 | 2.4434 | -0.045 | -0.992 | 0.322 | 0.776 | 1.288 |
| Statins | -0.1421 | 1.6565 | -0.005 | -0.086 | 0.932 | 0.479 | 2.086 |
| intercept | 72.8819 | 3.5943 |  | 20.277 | 0.000 |  |  |
| baPWV | -0.0071 | 0.0025 | -0.138 | -2.832 | 0.005 | 0.675 | 1.481 |
| Age | -0.2382 | 0.0631 | -0.186 | -3.776 | 0.000 | 0.667 | 1.499 |
| Female | 5.2721 | 1.2231 | 0.176 | 4.310 | 0.000 | 0.963 | 1.039 |
| CKD | -6.8963 | 3.3937 | -0.085 | -2.032 | 0.043 | 0.918 | 1.089 |
| CCB | -5.6229 | 1.3619 | -0.181 | -4.129 | 0.000 | 0.839 | 1.191 |
| βB | -3.8419 | 1.4599 | -0.115 | -2.632 | 0.009 | 0.838 | 1.194 |
| intercept | 69.6498 | 15.6213 |  | 4.459 | 0.000 |  |  |
| baPWV | -0.0122 | 0.0035 | -0.237 | -3.529 | 0.001 | 0.603 | 1.660 |
| Age | -0.2304 | 0.1010 | -0.159 | -2.281 | 0.023 | 0.563 | 1.778 |
| Female | 7.6626 | 2.0608 | 0.261 | 3.718 | 0.000 | 0.552 | 1.812 |
| CKD | -7.2657 | 5.7974 | -0.073 | -1.253 | 0.211 | 0.808 | 1.237 |
| CCB | -4.5148 | 1.9227 | -0.139 | -2.348 | 0.020 | 0.774 | 1.292 |
| βB | -3.6590 | 2.1903 | -0.103 | -1.671 | 0.096 | 0.717 | 1.395 |
| Hb | 0.5510 | 0.6779 | 0.060 | 0.813 | 0.417 | 0.495 | 2.019 |
| HbA1c | -0.8055 | 0.9208 | -0.053 | -0.875 | 0.383 | 0.757 | 1.321 |
| GFR | -0.0100 | 0.0464 | -0.013 | -0.216 | 0.829 | 0.722 | 1.385 |
| LDL | 0.0075 | 0.0216 | 0.019 | 0.349 | 0.727 | 0.901 | 1.110 |
| HDL | 0.0754 | 0.0713 | 0.063 | 1.058 | 0.291 | 0.774 | 1.293 |
| TG | 0.0034 | 0.0114 | 0.017 | 0.299 | 0.765 | 0.816 | 1.225 |
| CRP | -1.0341 | 2.4588 | -0.023 | -0.421 | 0.674 | 0.908 | 1.102 |
| intercept | 79.1534 | 9.5799 |  | 8.262 | 0.000 |  |  |
| baPWV | -0.0064 | 0.0027 | -0.131 | -2.348 | 0.019 | 0.653 | 1.531 |
| Age | -0.1918 | 0.0736 | -0.158 | -2.606 | 0.010 | 0.554 | 1.807 |
| Female | 6.6725 | 1.3977 | 0.227 | 4.774 | 0.000 | 0.891 | 1.122 |
| CKD | -8.3724 | 3.9478 | -0.100 | -2.121 | 0.035 | 0.916 | 1.092 |
| CCB | -5.1356 | 1.5418 | -0.162 | -3.331 | 0.001 | 0.852 | 1.174 |
| βB | -4.4672 | 1.6436 | -0.133 | -2.718 | 0.007 | 0.848 | 1.179 |
| LVEF | -0.2130 | 0.1026 | -0.096 | -2.077 | 0.039 | 0.948 | 1.055 |
| LVMI | 0.0543 | 0.0330 | 0.081 | 1.646 | 0.101 | 0.827 | 1.209 |
| septal e' | 32.2267 | 36.7863 | 0.057 | 0.876 | 0.382 | 0.477 | 2.098 |
| e/e' | -0.3970 | 0.3232 | -0.077 | -1.228 | 0.220 | 0.508 | 1.968 |
| intercept | 87.8017 | 6.8832 |  | 12.756 | 0.000 |  |  |
| baPWV | -0.0079 | 0.0025 | -0.161 | -3.130 | 0.002 | 0.683 | 1.464 |
| Age | -0.2071 | 0.0620 | -0.170 | -3.342 | 0.001 | 0.700 | 1.429 |
| Female | 6.1025 | 1.2432 | 0.213 | 4.909 | 0.000 | 0.961 | 1.041 |
| CKD | -7.8152 | 3.8432 | -0.090 | -2.034 | 0.043 | 0.926 | 1.080 |
| CCB | -5.5796 | 1.4226 | -0.179 | -3.922 | 0.000 | 0.865 | 1.156 |
| βB | -4.5555 | 1.5014 | -0.139 | -3.034 | 0.003 | 0.860 | 1.163 |
| LVEF | -0.2393 | 0.0912 | -0.113 | -2.623 | 0.009 | 0.980 | 1.020 |

HRR2 heart rate recovery at 2minute; baPWV, brachial-ankle pulse wave velocity; SE, standard error; VIF, variance inflation factor; BMI, body mass index; HTN, hypertension; DM, diabetes mellitus; CAD, coronary artery disease; CKD, chronic kidney disease; RAS, renin-angiotensin system; CCB, calcium channel blocker; βB, β-blocker; Hb, hemoglobin; GFR, glomerular filtration rate; LDL-C, low-density lipoprotein cholesterol; HDL-C, high-density lipoprotein cholesterol; TG, triglyceride; CRP, C-reactive protein; LVEF, left ventricular ejection fraction; LVMI, left ventricular mass index.
